# ACI/EG eutectic mixture mediated synthesis, characterization and *in vitro* osteoblast differentiation assessment of spiropyrrolo[1,2-*b*]isoquinoline analogues†

**DOI:** 10.1039/c8ra00646f

**Published:** 2018-05-02

**Authors:** Govindasami Periyasami, Natarajan Arumugam, Mostafizur Rahaman, Raju Suresh Kumar, Muthurangan Manikandan, Musaad A. Alfayez, Dhanaraj Premnath, Ali Aldalbahi

**Affiliations:** Department of Chemistry, College of Science, King Saud University P.O. Box 2455 Riyadh 11451 Saudi Arabia aaldalbahi@ksu.edu.sa pkandhan@ksu.edu.sa; Department of Organic Chemistry, University of Madras, Guindy Campus Chennai 600 025 India; Stem Cell Unit, Department of Anatomy, College of Medicine, King Saud University Riyadh Saudi Arabia; Department of Oral Biology Dentistry Building-D325 Winnipeg Manitoba Canada

## Abstract

An eco-friendly acetylcholine iodide–ethylene glycol (ACI/EG) deep eutectic mixture mediated green protocol has been developed for the synthesis of hitherto unexplored multi-functionalized linear tricyclic spiropyrrolo[1,2-*b*]isoquinoline analogues. The effects of the synthesized compounds on the osteoblast differentiation of hBMSC-TERT cell lines were investigated and promising results were observed with significant IC_50_ values. In addition, molecular modeling simulations were also performed with the 3D structure of BMP-2 to reveal binding interactions and orientations of highly potent spiropyrrolo[1,2-*b*]isoquinoline analogues.

## Abstract 2

An eco-friendly acetylcholine iodide–ethylene glycol (ACI/EG) deep eutectic mixture mediated green protocol has been developed for the synthesis of unexplored multi-functionalized linear tricyclic spiropyrrolo[1,2-*b*]isoquinoline analogues.

## Graphical abstract

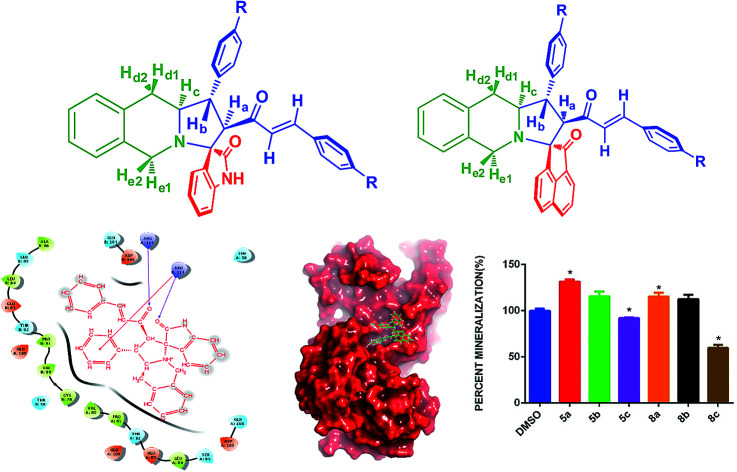

## 1. Introduction

Healthy bones are important for a safe and happy life. Bone growth is controlled by cells called osteoclasts (OCs) and osteoblasts (OBs) during skeletal development throughout the human life. Removal of mineralized bone by OCs followed by the formation of the bone matrix through the OBs is the systematic approach taken during bone remodeling. There are various factors that control bone remodeling including insulin-like growth factors (IGFs), tumor growth factor-beta (TGF-β), bone morphogenetic proteins (BMPs) and cytokines.^1^ Among the various inductive growth factors of bone morphogenetic proteins (BMPs) including kidney development, limb formation, angiogenesis, tissue fibrosis and tumor development, osteogenic differentiation is one of the well-known and notable regulating processes that involve BMP-2.^2^ It increases the number of mature OBs by enhancing the differentiation capability of various stem cells. Also, biological signal pathways for inducing osteogenic differentiation are regulated by the binding of BMP-2 with microdomains on cell surfaces.^3^ Thus, BMP-2 is a potent inducer of bone formation and it also enhances the efficiency of osteogenic differentiation. Lee *et al.*^4^ demonstrated that isoquinoline alkaloids induce OB differentiation of stromal cells without the loss of cell viability. But, to date, there is no report in the literature on the effects of pyrroloisoquinoline and its analogues on osteogenic differentiation.

**Fig. 1:**
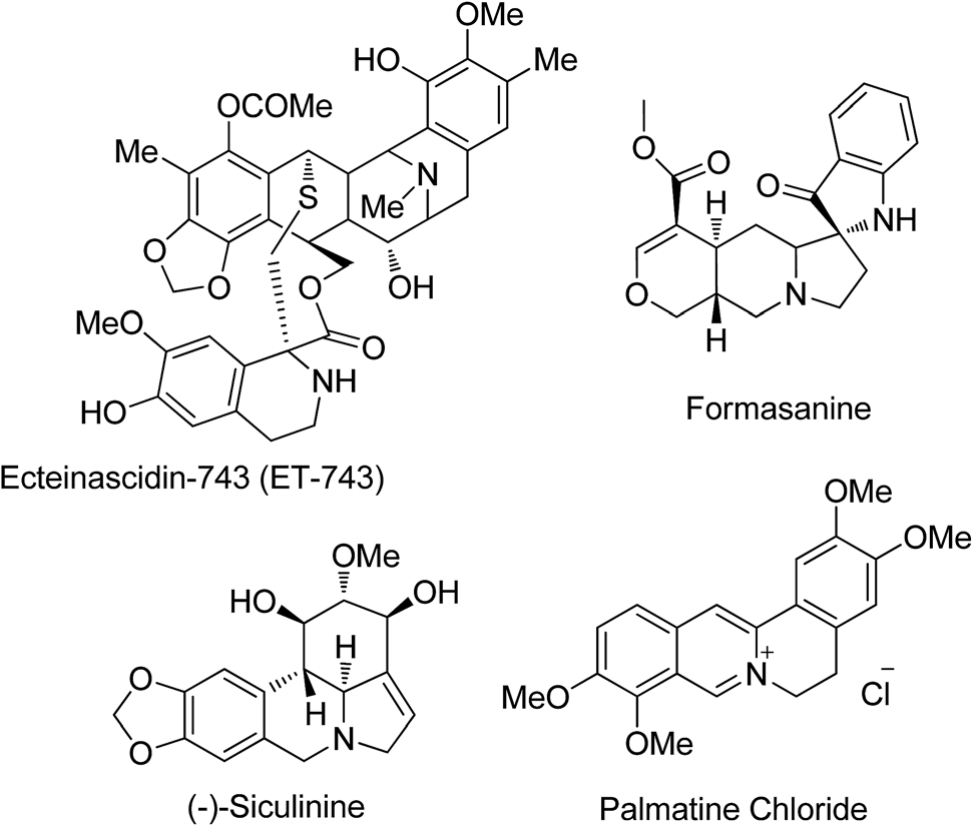
Pyrrolo[1,2-*b*]isoquinoline core and spiro-center natural products.

The pyrroloisoquinoline structural framework is present in a number of alkaloids including the ecteinascidin family, and cactus and *Hippeastrum* genus alkaloids (Fig. 1), and it has remarkable activities in the central nervous system^5^ besides possessing antitubercular,^6*a*^ antihypertensive,^6*b*^ anti HIV-1,^6*c*^ anti-leukemic,^6*d*^ and anti-cancer activities.^6*e*^ Its analogues can be used as radiotracers in positron emission tomography (PET) for imaging serotonin uptake sites.^7^ The tendency of lycorine alkaloids to inhibit cell growth and cell division is utilized to study their activity against a number of cancer cell lines, showing a decrease in tumor cell growth and an increase in survival rates with no observable adverse effects in animal treatment. Isoquinoline analogues containing spiro centers are frequently encountered in natural products and they possess diverse pharmacological properties.^8^ Also the spiro structural motif, including polycyclic spiro heterocycles, which intercalates easily by its nature into DNA, can be used as a class of effective anti-tumor pharmacophore units.^9^ The structural rigidity and complexity of spiro compounds are highly favorable for increasing affinity towards proteins by reducing the loss of conformational entropy, which is a topic of interest in drug discovery research.^10^

Our research aim is to synthesize spiropyrrolo[1,2-*b*]isoquinolines and study their effects on OB differentiation, particularly the significant increase in alkaline phosphatase activity (ALP) of ecteinascidin-743 (alkaloid in the ecteinascidin family), a marker of osteoblastic differentiation.^11^

## 2. Results and discussion

### 2.1. Chemistry

**Fig. 2:**
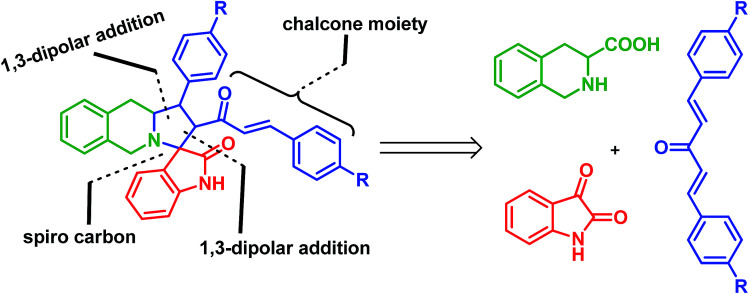
Synthetic strategy for linear tricyclic spiropyrrolo[1,2-*b*]isoquinoline cycloadducts.

Interesting synthetic methodologies have been reported in the literature for the synthesis of various pyrrolo[1,2-*a*]isoquinoline derivatives including one-pot or multi-step reactions including solid phase supported,^12*a*^ photoredox-catalyzed,^12*b*^ metal-catalyzed,^12*c*^ acid/base catalyzed,^12*d*^ and Pictet–Spengler cyclization^12*e*^ reactions. However, the syntheses of linear tricyclic spiropyrrolo[1,2-*b*]isoquinoline ring systems and their biological applications are relatively unexplored. The results of our recent report^13^ on the multicomponent reaction of an inexpensive quaternary alkylammonium class of eutectic mixture mediated heterocyclic molecules encouraged us to synthesize spiropyrrolo[1,2-*b*]isoquinoline analogues using a similar methodology. An inexpensive room temperature eutectic mixture,^14^ acetylcholine iodide–ethylene glycol (ACI/EG) mediated synthesis of spiro heterocycles, particularly pyrrolo[1,2-*b*]isoquinoline analogues, through azomethine ylide 1,3-dipolar cycloaddition reactions is yet to be reported. The retrosynthetic strategy explains the construction of regioselective spiropyrrolo[1,2-*b*]isoquinoline molecules from easily available starting materials in a single synthetic step as shown in Fig. 2.

During the synthesis, the chalcone group is retained in the spiro adduct which may be due to the stability of the mono adduct and to avoid the formation of the sterically hindered bis cycloadduct. One of the C=C bonds in the dipolarophiles, (1*E*,4*E*)-1,5-*p*-substituted diphenylpenta-1,4-dien-3-one derivatives,^15^ may preferably undergo an addition reaction with an azomethine ylide which leads to the unreacted prop-2-en-1-one unit in spiro heterocyclic hybrids. Spiropyrroloisoquinoline with a chalcone group is an excellent candidate in medicinal chemistry particularly in Alzheimer’s disease treatment,^16^ and it is also capable of inducing apoptosis^17^ and uncoupling mitochondrial respiration.^18^ In general, chalcone compounds did not show genotoxic effects and they may be devoid of this significant side effect.^19^

**Scheme 1:**
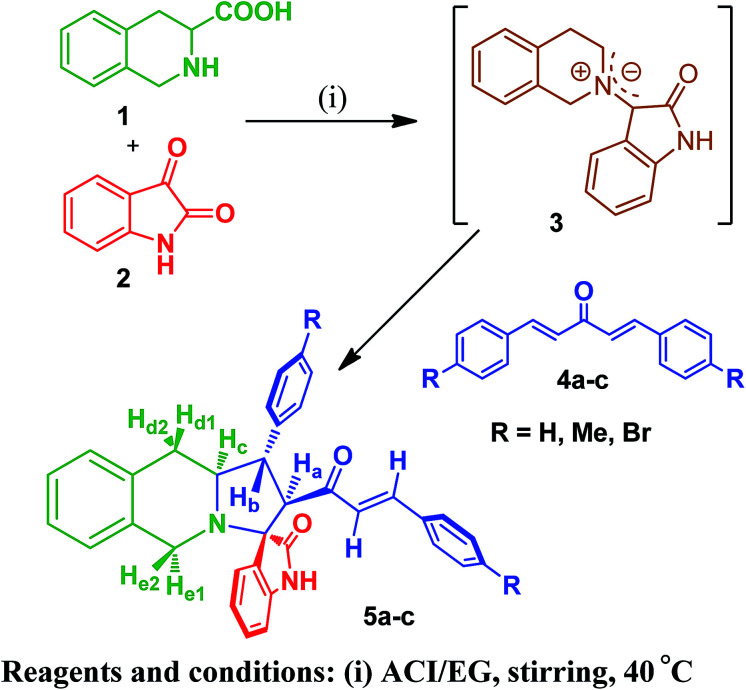
Synthesis of oxindole substituted monospiropyrrolo[1,2-*b*]isoquinolines 5a–c.

The spiro heterocycle tethered pyrrolo[1,2-*b*]quinoline analogues were obtained in good to excellent yields (85–92%) through 1,3-dipolar cycloaddition reactions of azomethine ylides generated *in situ* from an equimolar amount of a cyclic amino acid (1), a diketone (2 or 6) and various substituted dipolarophiles (4a–c) in ACI/EG at 40 °C. The required ACI/EG eutectic mixture, as a reaction medium, was prepared by mixing acetylcholine iodide and ethylene glycol in a 1 : 9 molar ratio and then the mixture was heated at 70 °C.^20^ Although our group has previously established and reported reaction conditions, we examined the synthesis of our target linear tricyclic spiropyrrolo[1,2-*b*]isoquinoline analogues under different reaction conditions (Table 1). As a result, it is noteworthy that the cycloaddition reaction in ACI/EG at 40 °C furnished an excellent yield of cycloadducts in 12 h (entry 8). Under the eutectic solvent medium, the interaction of ACI/EG *via* hydrogen bonding with the carbonyl group of the substrates, namely isatin/(1*E*,4*E*)-1,5-diphenylpenta-1,4-dien-3-one, would enhance the polarity of the carbon and oxygen. As a result, the electrophilicity of the isatin carbonyl would increase which in turn may influence the nucleophilic attack by the NH group of isoquinoline-3-carboxylic acid, then the subsequent dehydration and decarboxylation furnishes the 1,3-dipolar azomethine ylide intermediate. Similarly, the polarized enol form of (1*E*,4*E*)-1,5-diphenylpenta-1,4-dien-3-one is presumably activated by the interaction of the eutectic mixture with the dipolarophile *via* hydrogen bonding and then this facilitates the addition of the azomethine ylide. During this catalytic process, the dipolarophile underwent facile interaction with the intermediate azomethine ylide yielded cycloadducts significantly. Under different temperatures and times of the reaction, the yield of the product did not change significantly compared to the reaction at 40 °C for 12 h. Hence, all of the subsequent reactions (Scheme 1) were carried out using the optimized reaction conditions. After completion of the reaction (monitored by TLC), the pure product was obtained by column chromatography.

**Table 1:** Solvent and reaction condition optimization for the synthesis of pyrrolo[1,2-*b*]isoquinoline 5a^a^

Entry	Solvent system	Time (h)	Yield (%)
1	Ethanol	48	Trace*
2	Methanol	48	Trace*
3	Methanol/H_2_O	48	Trace*
4	Acetonitrile	48	Trace*
5	Acetonitrile/Et_3_N	48	15
6	Dioxane/MeCN (1 : 1)/Et_2_NH	48	10
7	ACI/EG, rt	48	60
**8**	**ACI/EG, 40 °C**	**12**	**90**

a General conditions: all reactions were performed under vigorous stirring at room temperature (except entry 8); optimized reaction conditions are in bold (entry 8); eutectic mixtures were subjected to high vacuum before use (entry 7 and 8); trace*: identifying product *R*_f_ by TLC.

ACI/EG was recovered from the reaction mixture by fractional distillation and dried overnight under high vacuum at 40 °C. It was further utilized for five consecutive reactions and the results revealed that the eutectic mixture is stable and recyclable without much significant loss of activity (Table 2).

**Table 2:** Reusability in various cycles of recovered ACI/EG in the synthesis of 5a at 40 °C

Medium	Yield percentage of isoxazolidine 5a in experiments				
	First	Second	Third	Fourth	Fifth
Recovered ACI/EG	90	88	88	87	86

**Fig. 3:**
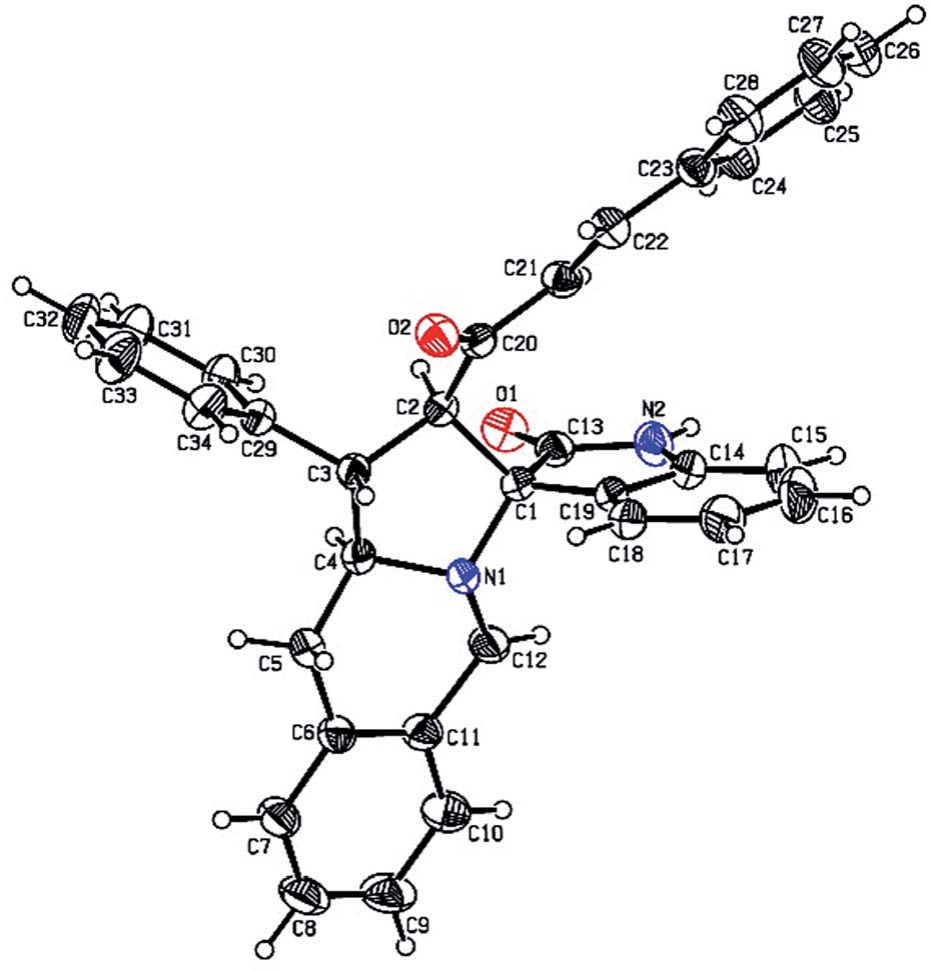
Oak Ridge Thermal Ellipsoid Plot (ORTEP) of cycloadduct 5a.

The FT-IR spectrum of compound 5a showed a band at 1673 cm^−1^ due to the α,β-unsaturated carbonyl and a band at 1686 cm^−1^ due to the amide carbonyl functional group. The NH group stretching was observed at 3382 cm^−1^. The ^1^H NMR spectrum of compound 5a exhibited a doublet at *δ* 4.19 (*J* = 9.0 Hz) for the H_a_ proton, a distorted doublet at *δ* 3.53 due to the benzylic proton and a multiplet at *δ* 3.95–4.01 due to the H_c_ proton. Two broad singlets observed at *δ* 2.89 and 3.94 corresponded to H_d_ and H_e_ protons, respectively. The *trans* vinyl protons of the olefinic system appeared as two doublets at *δ* 6.12 (*J* = 16.2 Hz) and *δ* 7.12 (*J* = 16.2 Hz). The *N*H proton of the oxindole ring resonated at *δ* 8.25 as a singlet. In the ^13^C NMR spectrum, the spiro quaternary carbon displayed a peak at 72.79 ppm. The oxindole carbonyl carbon was observed at 180.78 ppm. The α,β-unsaturated carbonyl carbon exhibited a peak at 196.06 ppm. A DEPT-135 study confirmed that the two methylene carbons appeared at 35.30 and 47.90 ppm. The mass spectrum of 5a exhibited a molecular ion peak at *m*/*z* 496.61 and the compound gave acceptable elemental analysis results. Finally, the structure and stereochemistry of cycloadduct 5a were elucidated unambiguously by single crystal X-ray diffraction analysis^21^ (Fig. 3). NMR spectra of all synthesized compounds are provided in the ESI.†

**Scheme 2:**
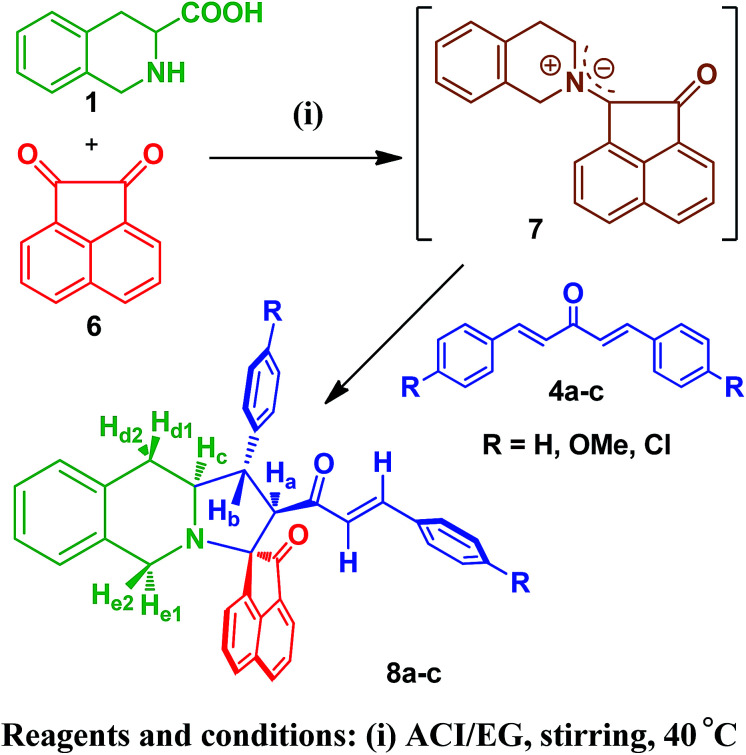
Synthesis of acenaphthoquinone substituted monospiropyrrolo[1,2-*b*]isoquinolines 8a–c.

To explore the synthetic utility of the optimized protocol, we have investigated the cycloaddition reaction with a different diketone *viz*, acenaphthoquinone as this structural motif possesses strong antioxidant properties, free radical scavenging activity and the ability to reduce lipid peroxidation.^22^ The generated azomethine ylide (7) readily undergoes cycloaddition with dipolarophiles (4a–c) affording the cycloadducts (8a–c) in good yields (Scheme 2).

**Scheme 3:**
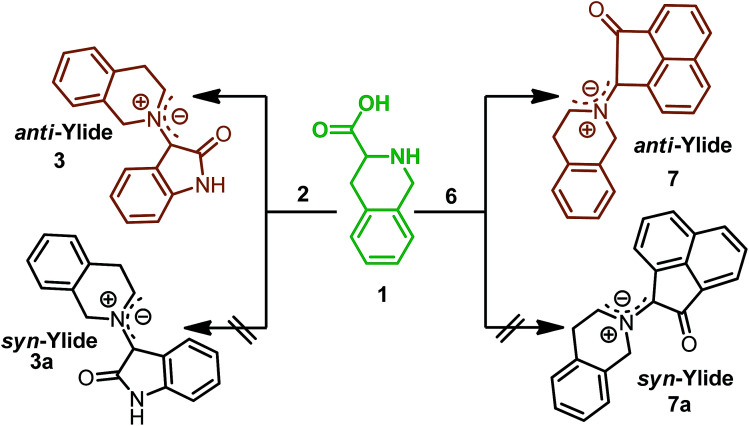
Formation of stable *anti*-ylides in the transition state.

The structure and regiochemistry of the cycloadducts (8a–c) were also unambiguously established by their spectroscopic and elemental analysis data.† The stability of formation of the azomethine ylide supports the relative stereochemistry of the synthesized novel spiropyrrolo[1,2-*b*]isoquinolines. In Schemes 1 and 2, formation of *syn*-ylides^23^3a and 7a may not be observed due to the unfavorable steric repulsion between the carbonyl group of the diketone and the isoquinoline ring. Most probably, *anti*-ylides^22^3 and 7, generated from tetrahydroisoquinoline-3-carboxylic acid, isatin (2) and acenaphthoquinone (6), respectively, are involved in the transition state which undergoes addition to dipolarophiles (4a–c) to give the observed cycloadducts (Scheme 3). In both Schemes 1 and 2, the cycloaddition proceeded *via* an *endo*-transition state^24^ and the possibility of formation of the other isomer *via* an *exo*-transition state was ruled out.

Under the optimized reaction conditions, stirring the ACI/EG medium at 40 °C, we obtained linear tricyclic spiropyrrolo[1,2-*b*]isoquinoline analogues in good yields. The yields and results are summarized in Table 3.

**Table 3:** Synthesis of linear tricyclic spiropyrrolo[1,2-*b*]isoquinoline analogues in ACI/EG medium by the reaction of amino acids, diketones and dipolarophiles through azomethine ylide 1,3-dipolar cycloaddition at 40 °C

Entry	Secondary amino acid	Diketone	Dipolarophile	Spiropyrrolo[1,2-*b*] isoquinoline^a^^,^^b^	Time (h)	Yield^c^ (%)
1	1	2	4a	5a	12	90
2	1	2	4b	5b	12	92
3	1	2	4c	5c	12	86
4	1	6	4a	8a	12	85
5	1	6	4b	8b	12	86
6	1	6	4c	8c	12	88

a Reaction conditions: vigorous stirring at 40 °C in ACI/EG eutectic mixture medium.

b Completion of the reaction monitored by TLC.

c Yield of the isolated product after flash column chromatography.

### 2.2. Biology

#### 2.2.1. Alizarin red S staining and osteoblast differentiation

**Fig. 4:**
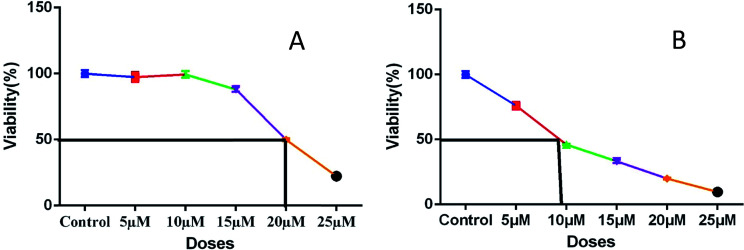
Plots (A) and (B) show IC_50_ values of representative compounds 5a (20 μM) and 8a (8 μM), respectively.

**Fig. 5:**
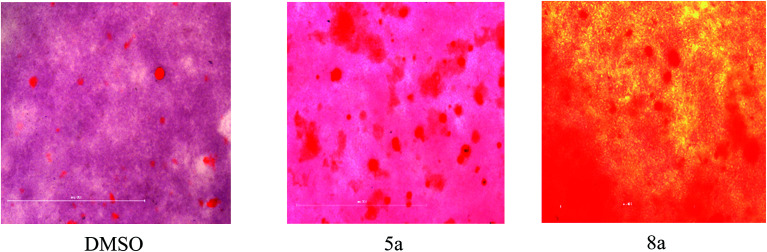
Qualitative analysis of alizarin red staining of mineralized nodule formation of 5a and 8a on day 7 in osteogenic differentiation media.

To evaluate the OB effect of newly synthesized spiropyrrolo[1,2-*b*]isoquinoline analogues, 5a and 8a were taken as representative compounds for each series, based on their IC_50_ values (Fig. 4; 5a = 20 μM; 8a = 8 μM). Initially, the mineralized matrix formation was confirmed by alizarin red S staining (Fig. 5).

**Fig. 6:**
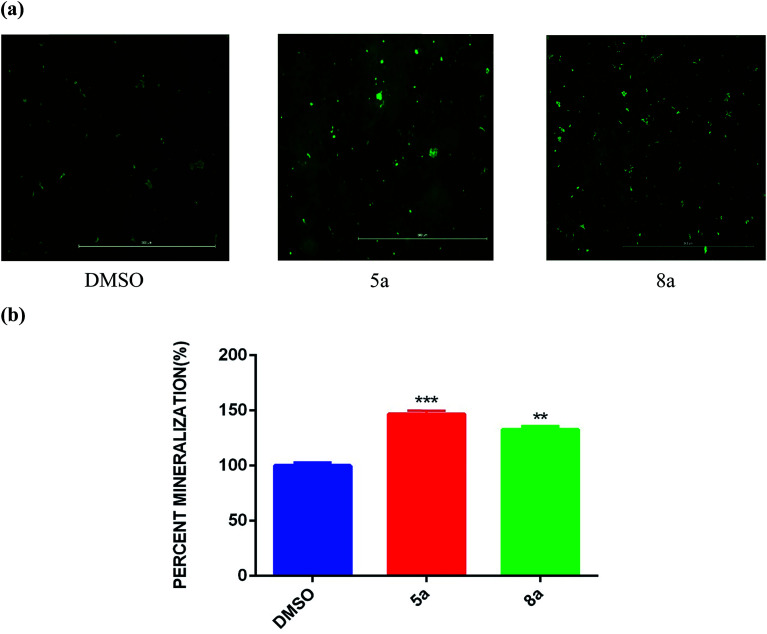
(a) Quantitative analysis of osteoimage mineralized nodule formation of 5a and 8a on day 7 in osteogenic differentiation media. (b) Quantitative mineralization of representative compounds 5a and 8a with DMSO as the standard {data are presented as the means ± SD from three independent experiments, *n* = 6; *p < 0.05; **p < 0.01, ***p < 0.001}.

In different concentrations of 5a at 15 μM and 8a at 5 μM, both the cycloadducts show significant effects on osteogenic differentiation of hBMSC-TERT (Fig. 5). Osteoimage staining of representative compounds was also carried out, and the observed nodule images (Fig. 6a) and the percentage of mineralization (Fig. 6b) are shown in Fig. 6.

#### 2.2.2. Osteoimage mineralization assay

**Fig. 7:**
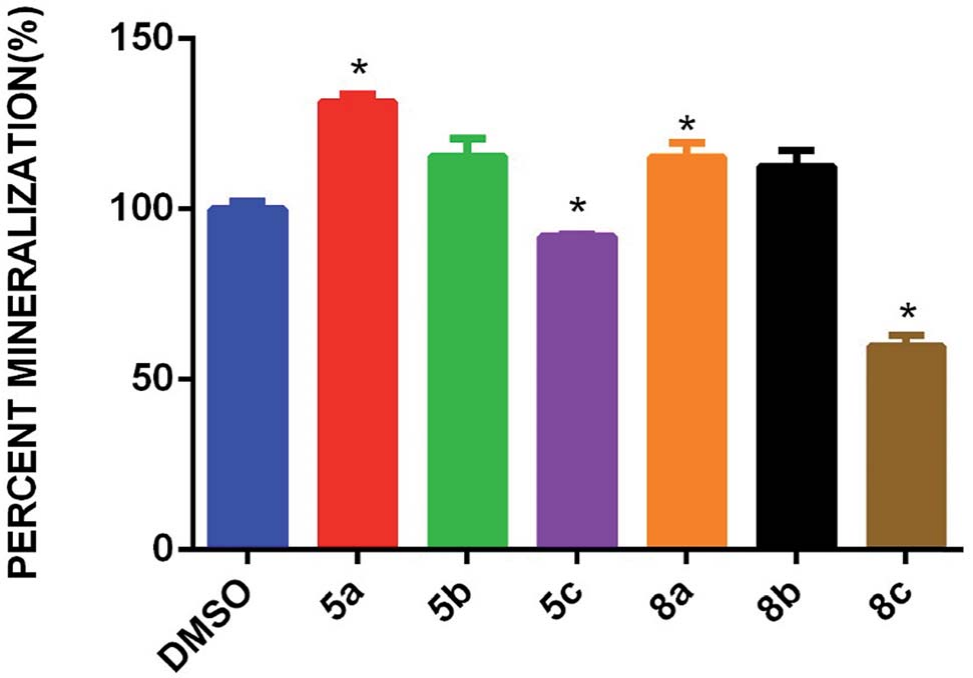
Osteoimage staining mineralization of spiropyrrolo[1,2-*b*]isoquinoline analogues 5a–c and 8a–c with DMSO as the standard. The data are presented as the means ± SD from three independent experiments, *n* = 6; *p < 0.05; **p < 0.01, ***p < 0.001.

Inspired by the initial promising OB differentiation activity of 5a and 8a with alizarin red S staining, we further assessed the OB differentiation by osteoimage staining to quantify the mineralized nodules that were induced by synthesized spiropyrrolo[1,2-*b*]isoquinoline analogues 5a–c and 8a–c. The results showed that significant mineralized nodules in the hBMSC-TERT cell line had formed by day 7 of osteogenic differentiation induction by the compounds. All of the isatin substituted spiropyrrolo[1,2-*b*]isoquinoline analogues 5a–c showed potent effects on OB differentiation, more than those of acenaphthoquinone substituted spiropyrrolo[1,2-*b*]isoquinoline analogues 8a–c. In both series, the unsubstituted group in the phenyl ring has a greater OB differentiation effect than the *p*-substituted derivatives. The accessible OB differentiation activities were observed for halogen substituted (4-bromo, 5c and 4-chloro, 8c) spiropyrrolo[1,2-*b*]isoquinolines. Also, the *para* substituted electron donating groups, *viz* methyl and methoxy in 5b and 8b, respectively, showed moderate OB differentiation effects compared to the unsubstituted phenyl ring which revealed that the electronic factors of the substituted groups may contribute to OB differentiation of hBMSC-TERT cell lines. In general, the good OB differentiation inducing effects of the synthesized spiropyrrolo[1,2-*b*]isoquinoline derivatives against hBMSC-TERT stem cells clearly shows that the synthesized cycloadducts are highly suitable for bone development. Though all of the synthesized compounds possessed significant OB differentiation inducing effects, spiropyrrolo[1,2-*b*]isoquinoline analogues with the isatin unit are more potent than their acenaphthoquinone analogues. Osteoimage quantification of spiropyrrolo[1,2-*b*]isoquinoline analogues 5a–c and 8a–c is presented in Fig. 7. The corresponding nodule images are presented in the ESI.†

### 2.3. Docking studies

**Fig. 8:**
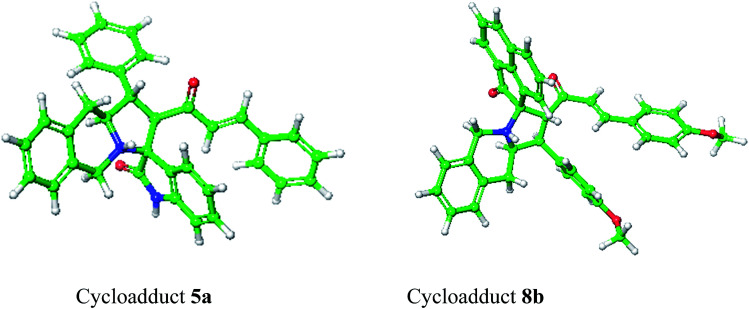
Optimized structure of cycloadducts 5a and 8b using the LigPrep module.

**Fig. 9:**
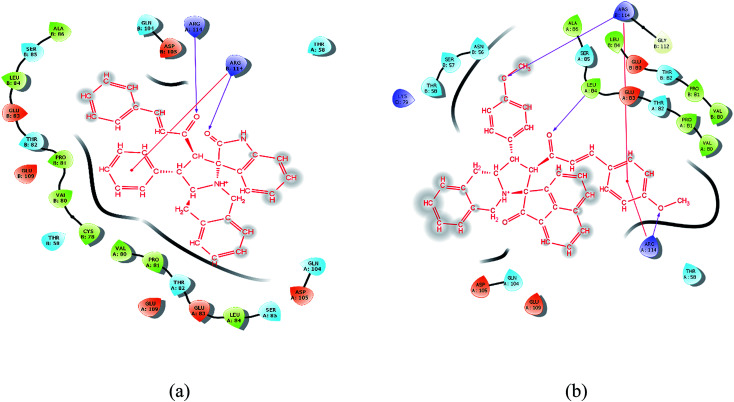
Orientation and binding interactions of (a) 5a with the BMP-2 active site and (b) 8b with the BMP-2 active site.

**Fig. 10:**
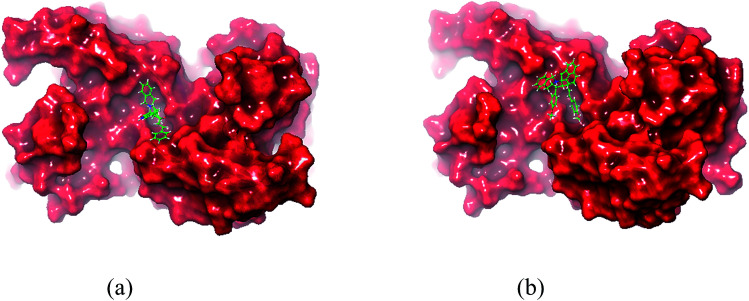
*In silico* packing illustration of representative compounds (a) 5a and (b) 8b with their appropriate binding packets of BMP-2.

Molecular docking study results of compounds 5a and 8b (Fig. 8) clearly reveal that the molecular interactions with the biologically proved active site of the BMP-2 protein with docking scores of −2.261 (5a) and −0.781 (8b) and the energy of −51.512 (**5a**) and −47.780 (8b). The molecular interaction studies demonstrate that ligands were bound on the same active site cavity in the molecule, as biologically proved and deposited in the scientific database. This is evident from the superposition of residues in active site molecules (12–114) and amino acid residues of the BMP-2 protein, in Fig. 9 and 10. At a distance of 4 Å, THR 58, ASN 56, SER 85, LEU 84, GLN 83, THR 82, PRO 81, VAL 80, ARG 114, GLN 109, ASP 105, and GLN 104 amino acids were found in the active sites and involved in biochemical interactions with compounds 5a and 8b. Docking of 5a with the BMP-2 protein is shown with two hydrogen bonding interactions with the respective active site molecules of ARG 114 (H–O–C) along with π–π stacking. Similarly 8b has also shown moderate hydrogen bonding interactions with the active site and has shown one hydrogen bonding interaction with ARG 114 with π–π stacking and another hydrogen bonding interaction with the LEU 84 amino acid. Compound 8b has shown a good hydrogen bonding backbone interaction with positively charged amino acids. Moreover, compound 5a has shown surface molecular interactions with strong hydrogen bonding with good energy scores. Computational docking study results reveal that the molecular biological activities shown are based on the molecular interaction and energy and structural orientation between the compounds and the active site amino acid polymer.

### 2.4. Evaluation of BMP-2 gene expression of spiropyrrolo[1,2-*b*]isoquinoline analogues

**Fig. 11:**
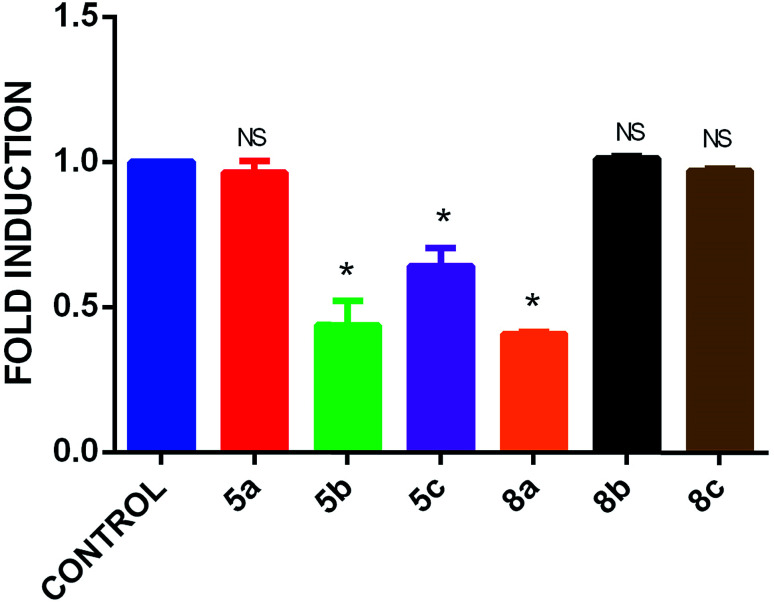
BMP-2 gene expression pattern of compounds 5a–c and 8a–c using qRT-PCR.

To authenticate the interactions between synthesized cycloadducts and the BMP-2 protein, based on the docking studies, the cycloadducts 5a–c and 8a–c have been subjected to BMP-2 expression evaluation after OS differentiation in OS differentiation media. In the spiroindolin-2-one series 5a–c, compound 5a maintained BMP-2 expression equal to the control, whereas 5b and 5c showed subdued expression. In the acenaphthylen-1-one series 8a–c, cycloadduct 8a resulted in lower expression of BMP-2, while 8b and 8c maintained BMP-2 expression level equal to the control. The results are presented in Fig. 11.

## 3. Conclusion

In conclusion, two series of spiropyrrolo[1,2-*b*]isoquinoline analogues were synthesized employing an ACI/EG eutectic mixture as an eco-friendly reaction medium through 1,3-dipolar cycloaddition methodology. To the best of our knowledge, this is the first report of the synthesis of spiropyrrolo[1,2-*b*]isoquinoline analogues in the ACI/EG benign reaction medium and their effects on OB differentiation of the hBMSC-TERT cell line. All of the synthesized compounds possessed the capability of enhancing the osteoblast differentiation of hBMSC-TERT cell lines with significant IC_50_ values of less than 20 μM. The structural and functional insights of spiropyrrolo[1,2-*b*]isoquinoline analogues with the BMP-2 protein were studied by molecular docking simulations using the Schrödinger Glide program. In addition, the hypothesis has been proved by aRT-PCR studies. These results emphasized the role of spiropyrrolo[1,2-*b*]isoquinolines in OB differentiation and could have myriad applications in bone biology.

## 4. Materials and methods

### 4.1. Cell culture

We employed hBMSC-TERT cell lines created from primary normal human bone marrow BMSCs by over-expressing the human telomerase reverse transcriptase gene (hTERT). We are grateful to our collaborator Prof. Moustapha Kassem, University of Odense, Southern Denmark for providing the hBMSC-TERT cell line.^25^ These cells have been extensively characterized and exhibit cellular and molecular phenotypes similar to those of primary hBMSCs and were cultured in Dulbecco’s modified Eagle’s medium (DMEM) supplemented with 4.5 g L^−1^d-glucose, 4 mM l-glutamine, 110 mg L^−1^ sodium pyruvate, 10% fetal bovine serum (FBS), 1× penicillin–streptomycin (Pen–Strep), and non-essential amino acids.

### 4.2. *In vitro* osteoblast differentiation

Cells were grown in 48-well plates in standard DMEM at a density of 1 × 10^5^ cells per mL. When cells reached 70–80% confluence, they were cultured in DMEM supplemented with osteogenic induction supplements containing 10% FBS, 1% Pen–Strep, 50 μg mL^−1^l-ascorbic acid (Wako Chemicals, Germany), 10 mM β-glycerophosphate (Sigma-Aldrich), 10 nM calcitriol (1α, 25-dihydroxyvitamin D3; Sigma), and 10 nM dexamethasone (Sigma-Aldrich). The medium was replaced three times per week. Compounds 5a and 8a were mixed with osteogenic differentiation media at 15 μM and 5 μM, respectively, to evaluate their osteogenic differentiation potential.

### 4.3. Cytochemical staining

#### 4.3.1. Alizarin red S staining of mineralized matrix

Cell layers were washed with PBS and then fixed with 4% paraformaldehyde (Sigma-Aldrich) for 15 min at room temperature. After removal of the fixative, each cell layer was rinsed with distilled water and then stained with 2% alizarin red S (Alizarin Red S Staining Kit; Cat. no. 0223; Sciencell Research Laboratories, Carlsbad, CA, USA) for 20–30 min at room temperature. Excess dye was washed away with water.

#### 4.3.2. Osteoimage mineralization assay

The mineralized matrix was quantified using the Osteoimage Mineralization Assay Kit (Cat. no. PA-1503; Lonza, Allendale, NJ, USA). After removal of the culture medium, cells were washed once with PBS and then fixed with 70% cold ethanol for 20 min. The appropriate amount, as recommended by the manufacturer, of diluted staining reagent was added, and the plates were incubated in the dark for 30 min at room temperature. Afterwards, the cells were washed, and the bound reagent was quantified using a fluorescence plate reader (excitation wavelength 492 nm; emission wavelength 520 nm).

### 4.4. Molecular docking

Molecular docking studies were performed using the Schrödinger Glide program (2017-4, Schrödinger LLC, New York 2014). The selected compounds 5a and 8b structures are drawn using ChemSketch software. In order to prepare ligand molecules with high quality, all-atom 3D structures for large numbers of drug-like molecules, starting with the 3D structures in SD Maestro format, LigPrep was used (Fig. 8). LigPrep produced a single, low-energy, 3D structure with corrected chiralities for each successfully processed input structure. The optimised ligands were docked into the bone morphogenetic protein-2 (BMP-2) structure using the Glide (SP & XP) module. The required typical structure file of BMP-2 protein structure was downloaded from the protein data bank (PDB). The structural file from experimental techniques may not be suitable for immediate use in molecular modelling calculations. PDB structures may miss information on connectivity that has been assigned along with bond orders and formal charges using the Protein Preparation Wizard. However, the receptor grid generation requires a “prepared” structure with all atoms and appropriate bond orders with formal charges. The shape and properties of receptor BMP-2 is generated on a grid by several different sets of fields around BMP-2 binding sites that provide progressively more accurate scoring of the ligand presence. To confirm the best molecular interaction and validate the docking score, the protocol was evaluated by re-docking.

### 4.5. Real time qRT-PCR

Total RNA was extracted from differentiating cells with a series of cycloadducts 5a–c and 8a–c using the innuPREP RNA Mini Kit (Cat no: 845-KS-2040250, Analytik Jena, Berlin, Germany) as recommended by the manufacturer. Total RNA was quantified using a Nanodrop spectrophotometer (Nanodrop 2000, Thermo Scientific). cDNA was synthesized from 1 μg of the RNA samples using a High Capacity cDNA Reverse Transcription kit (Applied Biosystems, Foster City, CA) using a ProFlex PCR System, Applied Biosystems according to the manufacturer’s instructions. Relative levels of mRNA were determined from cDNA using real time PCR (Applied Biosystem-Real Time PCR Detection System) with a Power SYBR Green PCR kit (Applied Biosystems, UK), according to the manufacturer’s instructions. Following normalisation to the reference gene *GAPDH*, quantification of gene expression was carried out using a comparative *C*_t_ method where Δ*C*_t_ is the difference between the *C*_t_ values of the target and reference gene. (Primer sequence: GAPDH – F 5′ CTGGTAAAGTGGATATTGTTGCCAT 3′, R 5′ TGGAATCATATTGGAACATGTAAACC 3′, BMP2 – F 5′ GGAACGGACATTCGGTCCTT 3′, R 5′ CACCATGGTCGACCTTTAGGA 3′).

## 5. Experimental

### 5.1. General procedure for the preparation of (1*E*,4*E*)-1,5-bis(*p*-substituted-phenyl)penta-1,4-dien-3-one, (4a–c)

A solution of *p*-substituted benzaldehyde (2.0 mol) and acetone (1.0 mol) was stirred at 15–20 °C in a 250 mL round bottom flask. 10% sodium hydroxide solution prepared and maintained at 15–20 °C was added slowly. The rate of addition was adjusted to make sure that the reaction temperature did not exceed 25–30 °C. The reaction mixture was vigorously stirred and then the precipitate was formed in 5 min. The stirring was continued for a further 1 hour. After completing the reaction as evidenced by TLC, dilute hydrochloric acid was added to neutralize the reaction mixture. The resulting precipitate was filtered and washed with cold water. The crude product was dried and recrystallized in an ethyl acetate/hexane mixture yielding 90–95% of pure (1*E*,4*E*)-1,5-bis(*p*-substituted-phenyl)penta-1,4-dien-3-one (4a–c).

### 5.2. General procedure for the synthesis of monospiropyrrolo[1,2-*b*]isoquinolines (5a–c)

A mixture of 1,2,3,4-tetrahydroisoquinoline-3-carboxylic acid (1) (1.2 equiv.), isatin (2) (1.2 equiv.) and (1*E*,4*E*)-1,5-bis(*p*-substituted-phenyl)penta-1,4-dien-3-one (4a–c) (1.0 equiv.) was stirred in an ACI/EG eutectic mixture for 1 hour. After the completion of the reaction as evidenced by TLC, in an adequate test, the eutectic mixture was removed by vacuum distillation and the residue was chromatographed over silica gel with the hexane–ethyl acetate mixture (8 : 2) to give novel monospiropyrrolo[1,2-*b*]isoquinolines (5a–c) in good to excellent yields.

#### 5.2.1. 3-Phenyl-4-[3′-phenyl-(*E*)-prop-2′-en-1′-one]spiro[5.3′]oxindolo-pyrrolo[1,2-*b*]isoquinoline, 5a


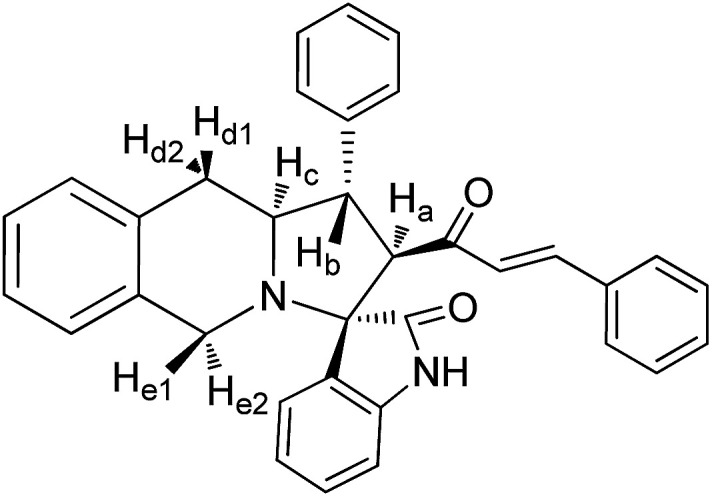
Yellow solid, mp 136 °C; yield: 90%; IR (KBr) 3382, 1686, 1673 cm^−1^; ^1^H (300 MHz, CDCl_3_) *δ* 2.89 (br s, 2H, H_d_), 3.53 (dis d, 1H, H_b_), 3.94 (br s, 2H, H_e_), 3.95–4.01 (m, 1H, H_c_), 4.19 (d, 1H, H_a_, *J* = 9.0 Hz), 6.12 (d, 1H, *J* = 16.2 Hz), 6.72–7.58 (m, 18H, ArH), 7.12 (d, 1H, *J* = 16.2 Hz), 8.25 (s, 1H, NH). ^13^C (75 MHz, CDCl_3_) 35.30, 47.90, 52.38, 62.42, 63.71, 72.79, 109.30, 123.39, 125.67, 126.15, 126.47, 126.87, 127.11, 127.18, 127.82, 128.04, 128.65, 128.73, 128.75, 129.03, 129.31, 130.35, 133.58, 133.76, 134.30, 139.89, 140.58, 142.46, 180.78, 196.06. EI-MS *m*/*z* 496.61 (M^+^). Anal. calcd for C_34_H_28_N_2_O_2_: C, 82.23; H, 5.68; N, 5.64%. Found: C, 82.31; H, 5.59; N, 5.71%.

#### 5.2.2. 3-(4′′-Methylphenyl)-4-[(3′-(4′′-methoxyphenyl))-(*E*)-prop-2′-en-1′-one]-spiro[5.3′]oxindolopyrrolo[1,2-*b*]isoquinoline, 5b


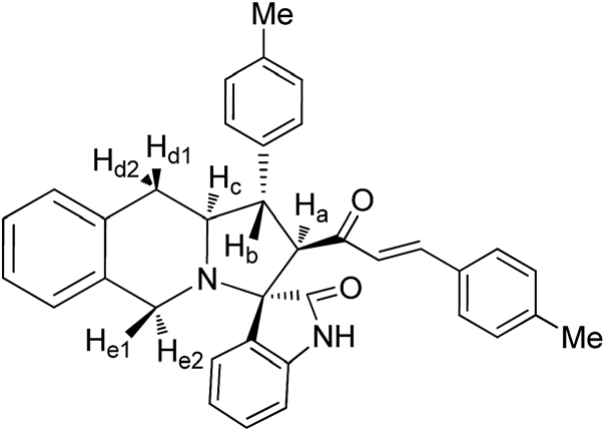
Yellow solid, mp 144 °C; yield: 92%; IR (KBr) 3384, 1686, 1674 cm^−1^; ^1^H (300 MHz, CDCl_3_) *δ* 2.81 (br s, 2H, H_d_), 3.16 (s, 3H, Me), 3.48 (dis d, 1H, H_b_), 3.91 (br s, 2H, H_e_), 3.96–4.10 (m, 1H, H_c_), 4.23 (d, 1H, H_a_, *J* = 9.0 Hz), 6.09 (d, 1H, *J* = 16.0 Hz), 6.64–7.53 (m, 16H, ArH), 7.14 (d, 1H, *J* = 16.0 Hz), 8.29 (s, 1H, NH). ^13^C (75 MHz, CDCl_3_) 29.81, 34.28, 39.64, 56.38, 61.31, 62.69, 72.22, 110.52, 122.38, 126.49, 126.52, 126.59, 126.76, 127.09, 127.27, 127.86, 127.99, 128.29, 128.64, 128.70, 129.41, 129.49, 130.51, 133.58, 133.69, 134.48, 138.92, 140.11, 142.43, 180.99, 197.60. EI-MS *m*/*z* 556.65 (M^+^). Anal. calcd for C_36_H_32_N_2_O_2_: C, 82.41; H, 6.15; N, 5.35%. Found: C, 82.66; H, 6.22; N, 5.28%.

#### 5.2.3. 3-(4′′-Bromophenyl)-4-[(3′-(4′′-bromophenyl))-(*E*)-prop-2′-en-1′-one]spiro[5.3′]oxindolopyrrolo[1,2-*b*]isoquinoline, 5c


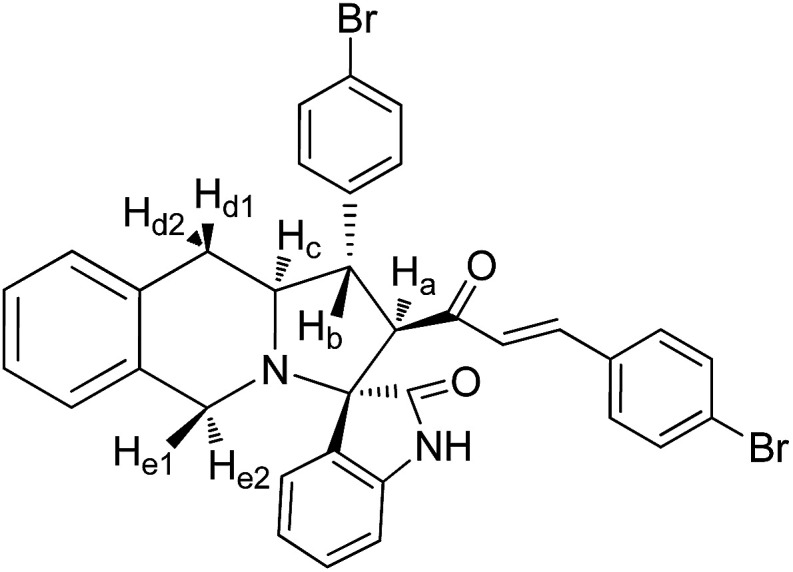
Pale yellow solid, mp 122 °C; yield: 86%; IR (KBr) 3382, 1683, 1676 cm^−1^; ^1^H (300 MHz, CDCl_3_) *δ* 2.88 (br s, 2H, H_d_), 3.51 (dis d, 1H, H_b_), 3.87 (br s, 2H, H_e_), 3.91–4.19 (m, 1H, H_c_), 4.35 (d, 1H, H_a_, *J* = 9.0 Hz), 6.21 (d, 1H, *J* = 16.2 Hz), 6.98–8.16 (m, 16H, ArH), 7.20 (d, 1H, *J* = 16.2 Hz), 8.16 (s, 1H, NH). ^13^C (75 MHz, CDCl_3_) 34.29, 39.02, 49.62, 58.31, 61.64, 70.22, 111.12, 121.00, 125.09, 126.31, 126.38, 126.86, 127.12, 127.89, 128.22, 128.29, 128.58, 128.66, 128.89, 129.39, 129.59, 129.91, 133.19, 133.64, 134.84, 138.89, 140.56, 142.73, 181.52, 197.85. EI-MS *m*/*z* 654.36 (M^+^). Anal. calcd for C_34_H_26_Br_2_N_2_O_2_: C, 62.40; H, 4.00; N, 4.28%. Found: C, 62.59; H, 3.89; N, 5.01%.

### 5.3. General procedure for the synthesis of monospiropyrroloisoquinolines (8a–c)

A mixture of 1,2,3,4-tetrahydroisoquinoline-3-carboxylic acid (1) (1.2 equiv.), acenaphthoquinone (6) (1.2 equiv.) and (1*E*,4*E*)-1,5-bis(*p*-substituted-phenyl)penta-1,4-dien-3-one (4a–c) (1.0 equiv.) was stirred in an ACI/EG eutectic mixture for 1 hour. After the completion of the reaction as evidenced by TLC, in an adequate test, the eutectic mixture was removed by vacuum distillation and the residue was chromatographed over silica gel with the hexane–ethyl acetate mixture (8 : 2) to give novel monospiropyrrolo[1,2-*b*]isoquinolines (8a–c) in good yields.

#### 5.3.1. 3-Phenyl-4-[3′-phenyl-(*E*)-prop-2′-en-1′-one]-spiro[5.2′]acenapthen-1′-ono-pyrrolo[1,2-*b*]isoquinoline, 8a


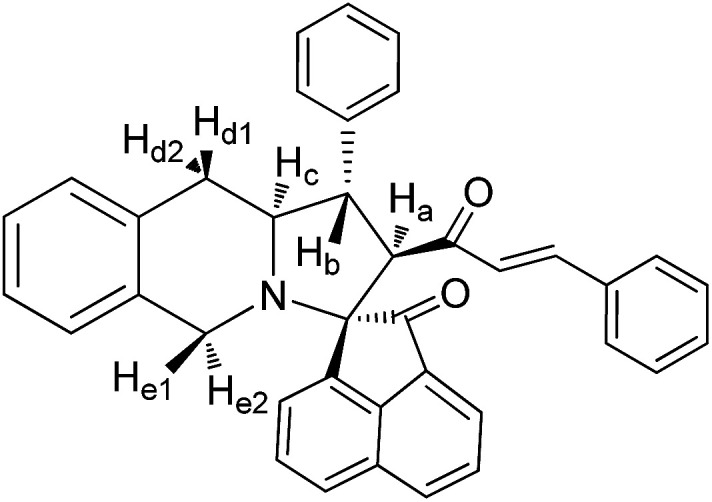
Yellow solid, mp 186 °C; yield: 85%; IR (KBr) 1716, 1678 cm^−1^; ^1^H (300 MHz, CDCl_3_) *δ* 2.93 (br s, 2H, H_d_), 3.42 (dis d, 1H, H_b_), 3.94 (d, 1H, H_a_), 4.08–4.09 (m, 2H, H_d_), 4.13–4.19 (m, 1H, H_c_), 5.69 (d, 1H, *J* = 16.2 Hz), 6.65–8.05 (m, 21H, ArH with alkene H). ^13^C (75 MHz, CDCl_3_) 35.44, 48.00, 52.67, 63.17, 64.25, 72.81, 121.09, 123.82, 125.02, 125.62, 126.13, 126.35, 126.42, 127.09, 127.67, 127.95, 128.44, 128.70, 128.75, 129.07, 129.12, 130.09, 130.23, 131.50, 132.44, 133.67, 133.78, 134.00, 137.64, 140.23, 141.84, 142.53, 196.42, 210.06. EI-MS *m*/*z* 531.64 (M^+^). Anal. calcd for C_38_H_29_NO_2_: C, 85.85; H, 5.50; N, 2.63%. Found: C, 85.91; H, 5.43; N, 2.70%.

#### 5.3.2. 3-(4′′-Methoxyphenyl)-4-[(3′-(4′′-methoxyphenyl))-(*E*)-prop-2′-en-1′-one]spiro[5.2′]acenapthen-1′-ono-pyrrolo[1,2-*b*]isoquinoline, 8b


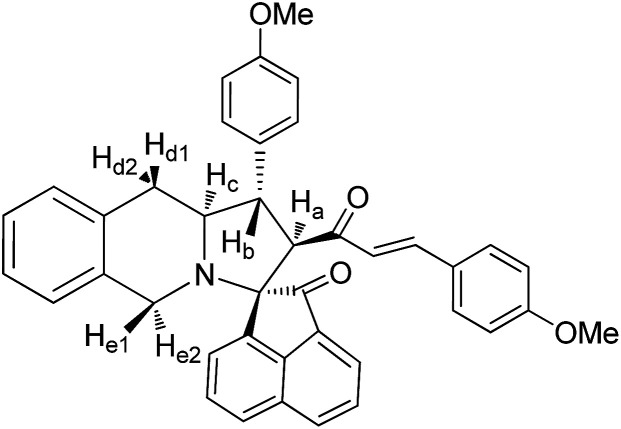
Colourless solid, mp 160 °C; yield: 86%; IR (KBr) 1716, 1678 cm^−1^; ^1^H (300 MHz, CDCl_3_) *δ* 2.96 (br s, 2H, H_d_), 3.13 (dis d, 1H, H_b_), 3.68 (s, 3H, OMe), 3.98 (d, 1H, H_a_), 4.00–4.03 (m, 2H, H_d_), 4.11–4.14 (m, 1H, H_c_), 5.55 (d, 1H, *J* = 16.2 Hz), 6.61–8.13 (m, 19H, ArH with alkene H). ^13^C NMR (75 MHz, CDCl_3_) 32.52, 46.21, 52.33, 56.62, 62.95, 64.19, 73.01, 120.85, 123.60, 125.42, 125.39, 125.90, 126.33, 126.44, 127.09, 127.67, 127.91, 128.49, 128.65, 128.71, 129.00, 129.09, 130.05, 130.53, 131.17, 132.46, 133.61, 133.68, 134.12, 137.66, 140.28, 141.68, 142.98, 196.19, 210.00. EI-MS *m*/*z* 591.69 (M^+^). Anal. calcd for C_40_H_33_NO_4_: C, 81.20; H, 5.62; N, 2.37%. Found: C, 81.26; H, 5.58; N, 2.42%.

#### 5.3.3. 3-(4′′-Chlorophenyl)-4-[(3′-(4′′-chlorophenyl))-(*E*)-prop-2′-en-1′-one]spiro[5.2′]acenapthen-1′-ono-pyrrolo[1,2-*b*]isoquinoline, 8c


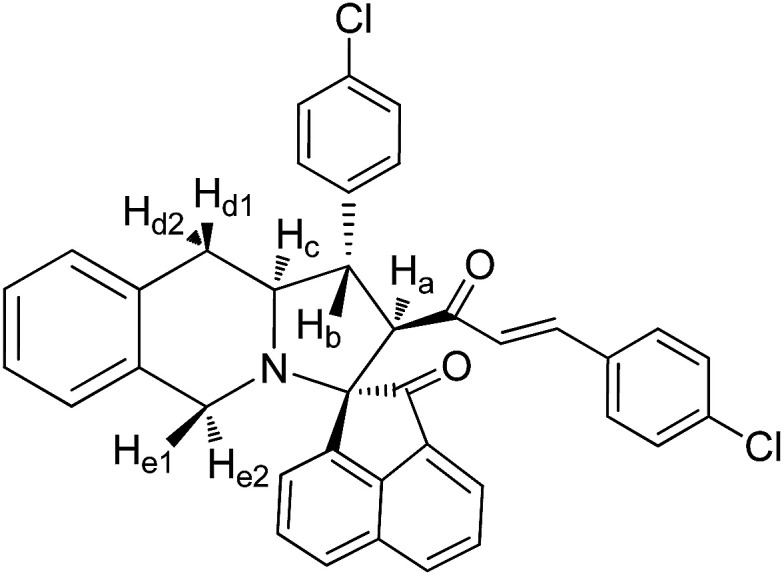
Colourless solid, mp 172 °C; yield: 88%; IR (KBr) 1720, 1679 cm^−1^; ^1^H (300 MHz, CDCl_3_) *δ* 2.98 (br s, 2H, H_d_), 3.32 (dis d, 1H, H_b_), 3.89 (d, 1H, H_a_), 4.01–4.03 (m, 2H, H_d_), 4.09–4.13 (m, 1H, H_c_), 5.62 (d, 1H, *J* = 16.2 Hz), 6.76–8.16 (m, 19H, ArH with alkene H). ^13^C NMR (75 MHz, CDCl_3_) 31.25, 45.95, 52.13, 62.91, 64.18, 73.09, 120.83, 123.44, 125.38, 125.54, 125.89, 126.30, 126.41, 126.98, 127.59, 127.88, 128.52, 128.64, 128.73, 129.07, 129.10, 130.00, 130.42, 131.24, 132.50, 133.61, 133.68, 134.17, 137.68, 140.30, 141.72, 142.96, 196.23, 210.15. EI-MS *m*/*z* 600.53 (M^+^). Anal. calcd for C_38_H_27_Cl_2_NO_2_: C, 76.00; H, 4.53; N, 2.33%. Found: C, 76.07; H, 4.59; N, 2.29%.

## Conflicts of interest

There are no conflicts to declare.

## Supplementary Material

RA-008-C8RA00646F-s001
