# Supplementary material for: ACI/EG eutectic mixture mediated synthesis, characterization and in vitro osteoblast differentiation assessment of spiropyrrolo[1,2-b]isoquinoline analogues
Source: RSC Adv. 2018 May 2;8(29):16303–13. doi: 10.1039/c8ra00646f (PMC9080264; doi:10.1039/c8ra00646f)

Electronic Supplementary Material (ESI) for RSC Advances.
This journal is © The Royal Society of Chemistry 2018

‡ Electronic Supplementary Information

ACI/EG eutectic mixture mediated synthesis, characterization and *in vitro* osteoblast differentiation assessment of spiropyrrolo[1,2-*b*]isoquinoline analogues

Govindasami Periyasami,*[ab] Natarajan Arumugam,[a] Mostafizur Rahaman,[a] Raju Suresh Kumar,[a] Muthurangan Manikandan,[c] Musaad A. Alfayez,[c] Dhanaraj Premnath,[d] Ali Aldalbahi,*[a]

[a] Department of Chemistry, College of Science, King Saud University, P.O. Box 2455, Riyadh 11451, Saudi Arabia.

[b] Department of Organic Chemistry, University of Madras, Guindy Campus, Chennai 600 025, India.

[c] Stem Cell Unit, Department of Anatomy, College of Medicine, King Saud University, Riyadh, Saudi Arabia

[d] Department of Oral Biology, Dentistry Building-D325, Winnipeg, Manitoba, Canada.

Corresponding authors E-mail: pkandhan@ksu.edu.sa, aaldalbahi@ksu.edu.sa

Table of contents

S. No.	**Content**	Page
1	General considerations	02
2	General procedure for preparation of dipolarophiles **3a-c**	02
3	General procedure for synthesis of mono spiro pyrroloisoquinolines **5a-c**	02
4	General procedure for synthesis of mono spiro pyrroloisoquinolines **8a-c**	03
5	Figure 1: ^{1}H-NMR spectrum of **5a**	04
6	Figure 2: ^{13}C-NMR spectrum of **5a**	05
7	Figure 3: DEPT-135 spectrum of **5a**	06
8	Figure 4: ^{1}H-NMR spectrum of **8a**	07
9	Figure 5: ^{13}C-NMR spectrum of **8a**	08
10	Figure 6: DEPT-135 spectrum of **8a**	09
11	Figure 7: Osteoimage staining of mineralized nodules (OIN) of **5a-c** & **8a-c**	10

General considerations

All melting points were uncorrected. IR spectra were recorded on a SHIMADZU IR-8300 series FT-IR spectrophotometer. ^1H NMR and ^{13}C NMR spectra were recorded on BRUKER 300 MHz instrument in $CDCl_3$ and DMSO-d_6 solvent with TMS as a standard. Mass spectra were recorded on a JEOL-DX303 HF mass spectrophotometer. Elemental analyses were carried out by Perkin-Elmer CHNS 2400B and Carlo Erba 1106 instruments. Single crystal X-ray diffraction analyses were performed by Bruker SMART APEX CCD area-detector diffractometer and Bruker SMART APEXII CCD area-detector diffractometer. Column chromatography was performed on silica gel (ACME, 100 -200 mesh). Routine monitoring of the reaction was made using thin layer chromatography developed on glass plates coated with silica gel-G (ACME) of 25 mm thickness and visualized with iodine.

1. General procedure for preparation of (1*E*, 4*E*)-1,5-bis(*p*-substituted-phenyl)penta-1,4-dien-3-one, (3a-c)

A solution of *p*-substituted benzaldehyde (2.0 mol) and acetone (1.0 mol) was stirred at 15 – 20 °C in a 250 mL round bottom flask. 10 % sodium hydroxide solution, was prepared and maintained at 15 – 20 °C, was added slowly. Adjust the rate of addition to maintain the reaction temperature not exceeding to 25-30 °C. The reaction mixture was vigorously stirred then the precipitate was formed in five minutes. The stirring was continued for further one hour. After completing the reaction as evidenced by TLC, dilute hydrochloric acid was added to neutralize the reaction mixture. The resulting precipitate was filtered and washed with cold water. The crude product was dried and recrystallize in ethyl acetate / hexane mixture yielded 90 - 95 % of pure (1*E*, 4*E*)-1,5-bis(*p*-substituted-phenyl)penta-1,4-dien-3-one (**3a-c**).

2. General procedure for synthesis of monospiropyrroloisoquinolines, (5a-c)

A mixture of 1,2,3,4-tetrahydroisoquinoline-3-carboxylic acid (**1**) (1.2 equiv), isatin (**2**) (1.2 equiv) and (1*E*,4*E*)-1,5-bis(*p*-substituted-phenyl)penta-1,4-dien-3-one (**3a-c)** (1.0 equiv) was stirred in ACI/EG ionic liquid for 12 hours. After the completion of reaction as evidenced by TLC, in adequate test, the ionic liquid was removed by vacuum distillation and the residue was

chromatographed over silica gel with hexane-ethyl acetate mixture (8:2) to give novel mono spiropyrrolo[1,2-b]isoquinolines (**5a-c**) in good to excellent yield.

3. General procedure for synthesis of monospiropyrroloisoquinolines, (8a-c)

A mixture of 1,2,3,4-tetrahydroisoquinoline-3-carboxylic acid (**1**) (1.2 equiv), acenaphthoquinone (**6**) (1.2 equ) and (1*E*, 4*E*)-1,5-bis(*p*-substituted-phenyl)penta-1,4-dien-3-one (**3a-c)** (1.0 equiv) was stirred in ACI/EG ionic liquid for 12 hours. After the completion of reaction as evidenced by TLC, in adequate test, the ionic liquid was removed by vacuum distillation and the residue was chromatographed over silica gel with hexane-ethyl acetate mixture (8:2) to give novel mono spiropyrrolo[1,2-b]isoquinolines (**8a-c**) in good yield.

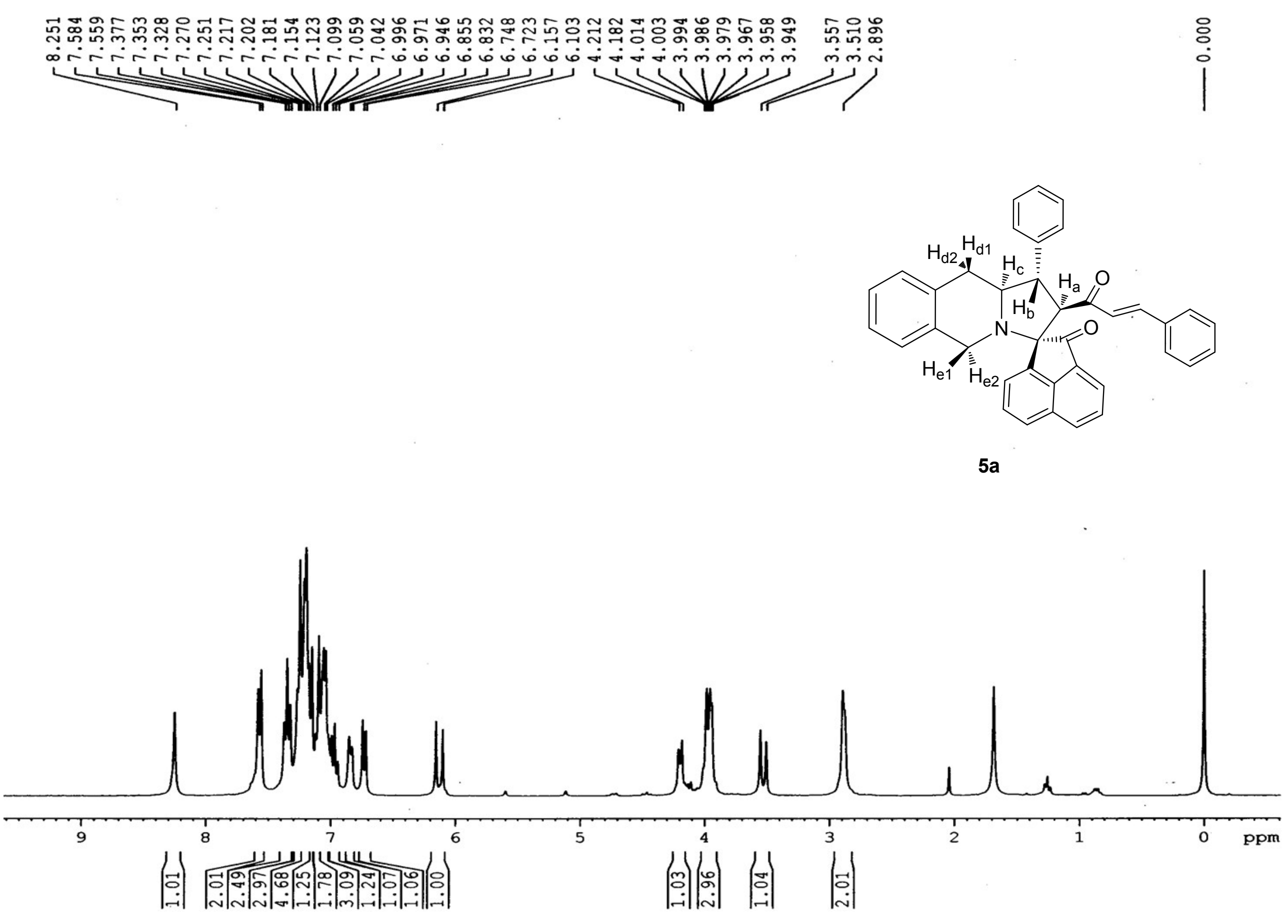

Figure 1: ^{1}H NMR spectrum of **5a**

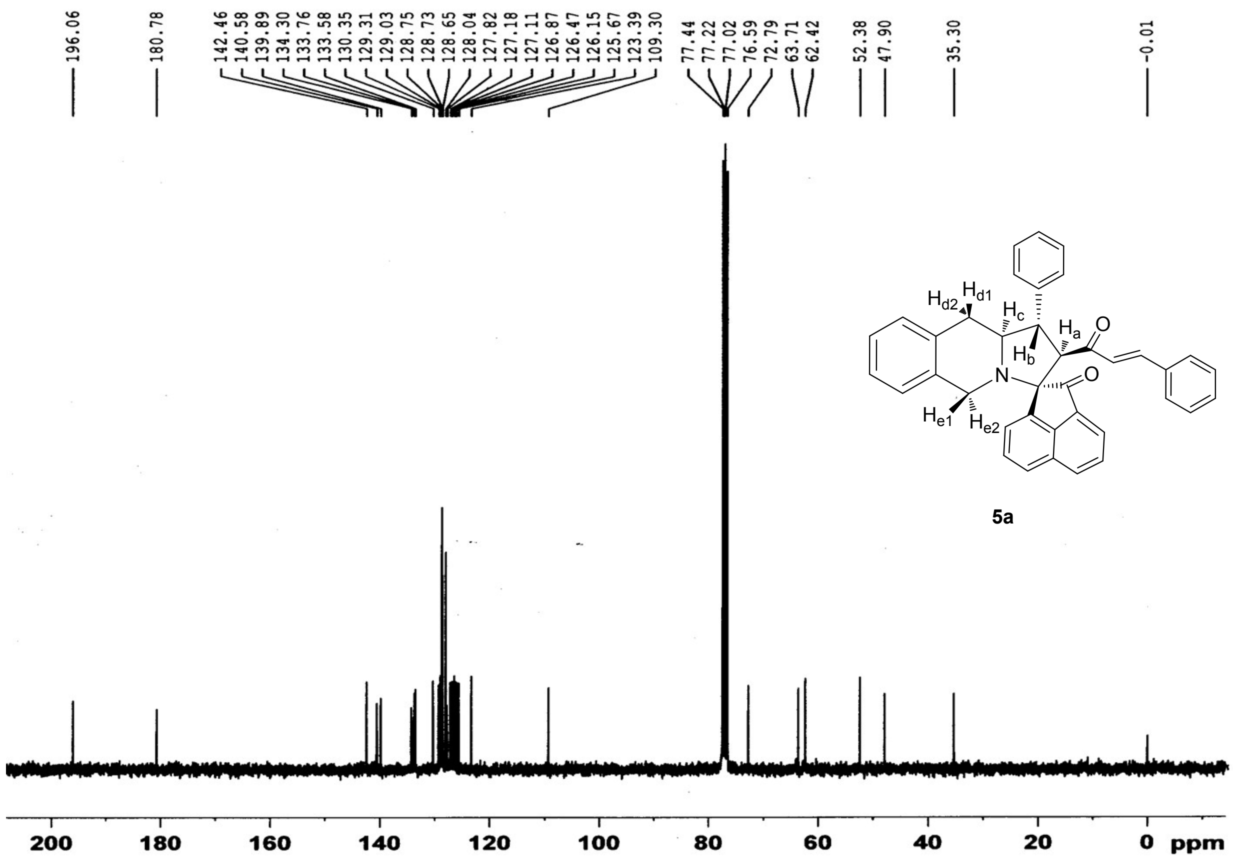

Figure 2: ^{13}C NMR spectrum of **5a**

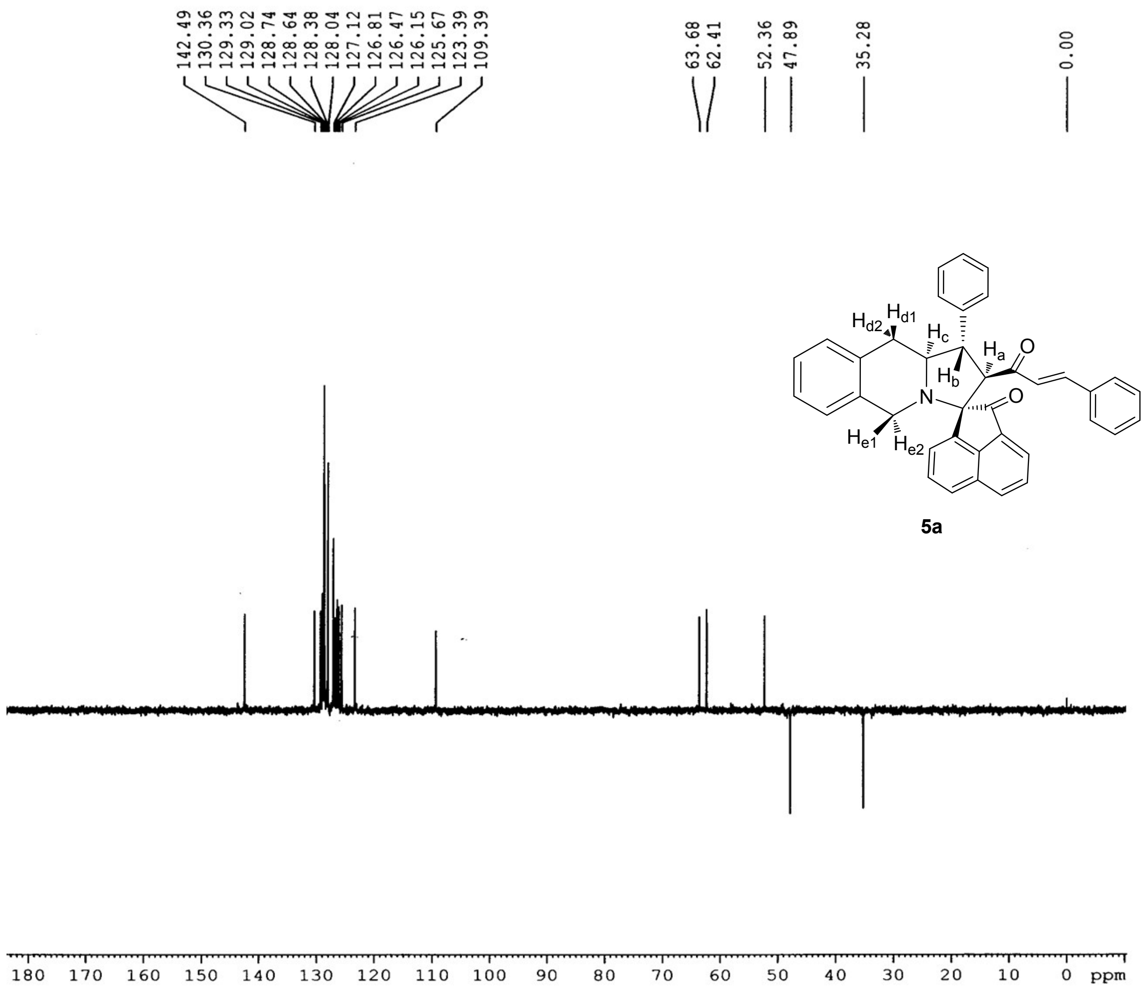

Figure 3: DEPT 135 spectrum of **5a**

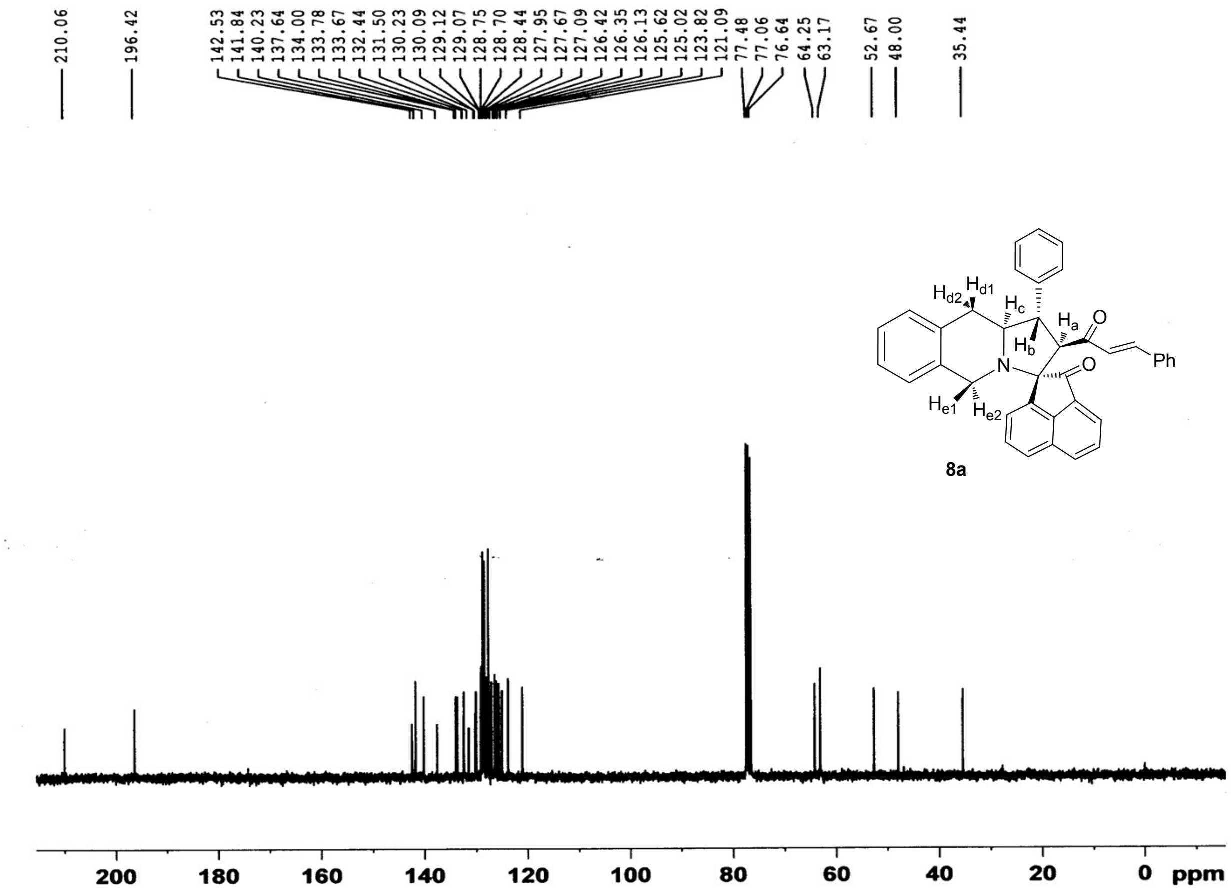

Figure 4: ^{1}H NMR spectrum of **8a**

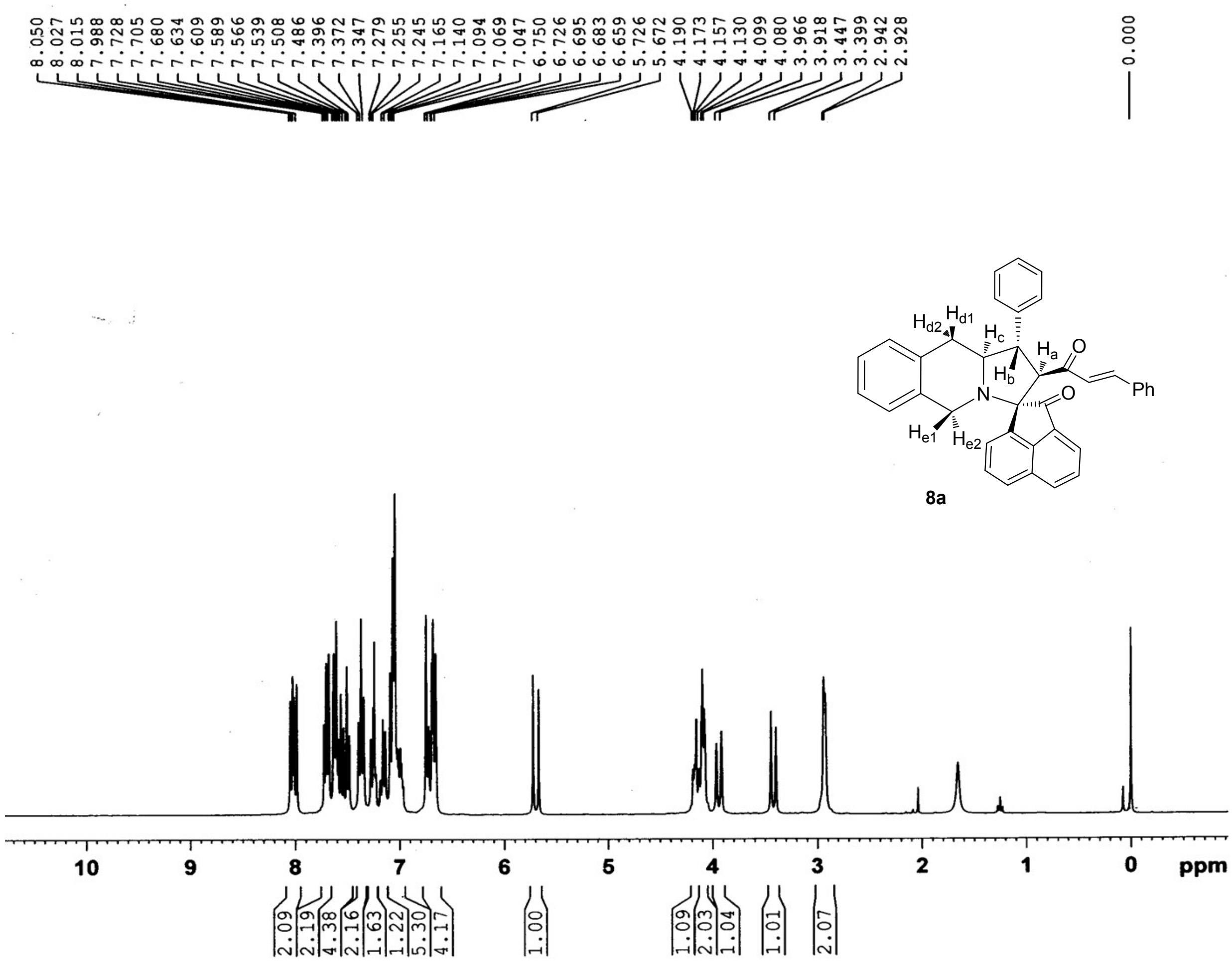

Figure 5: ^{13}C NMR spectrum of **8a**

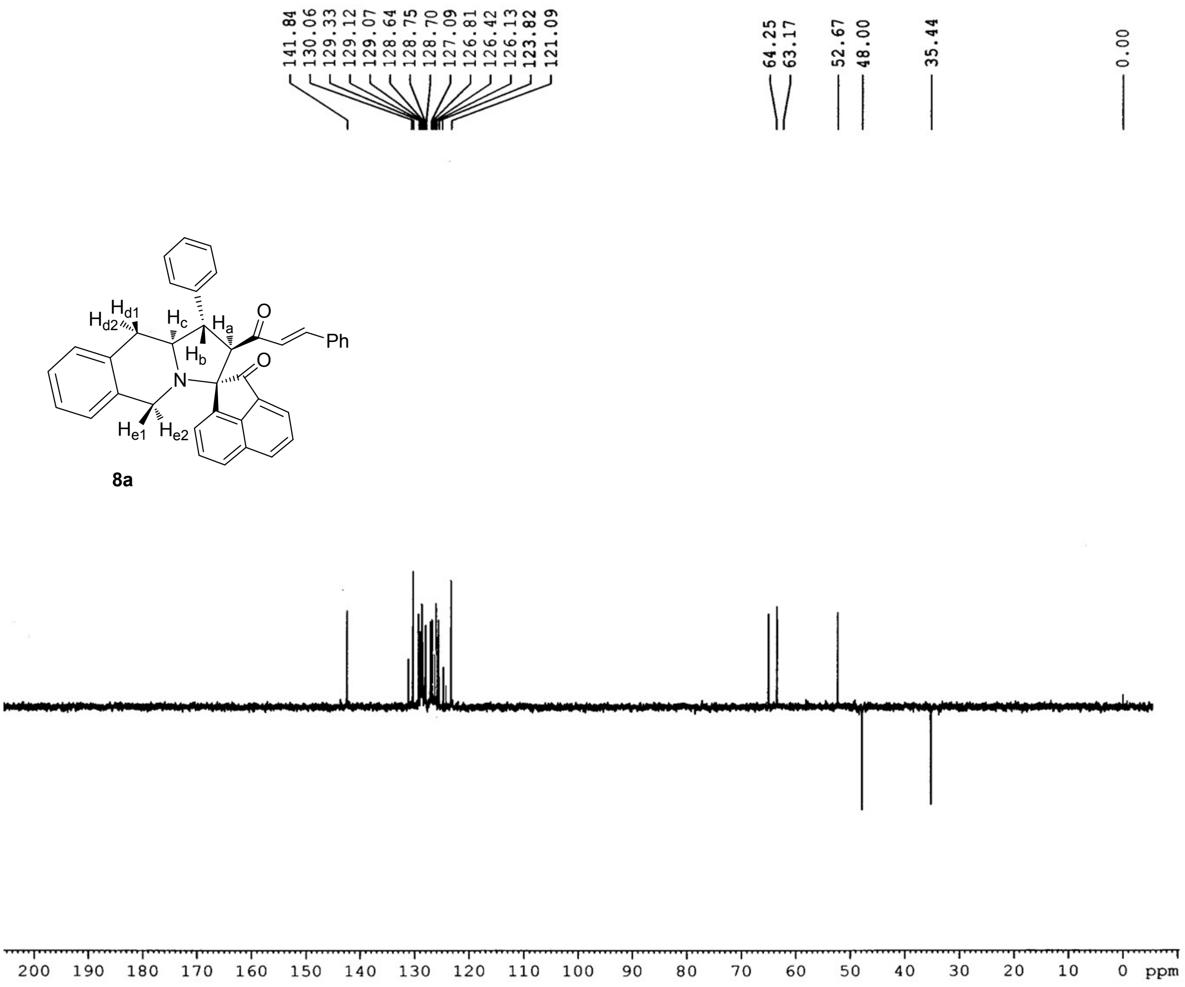

Figure 6: DEPT-135 spectrum of **8a**

Figure 7. Osteoimage staining of mineralized nodules (OIN) of spiropyrrolo[1,2-*b*]isoquinolines **5a-c** and **8a-c** at day 7 in osteogenic differentiation media.

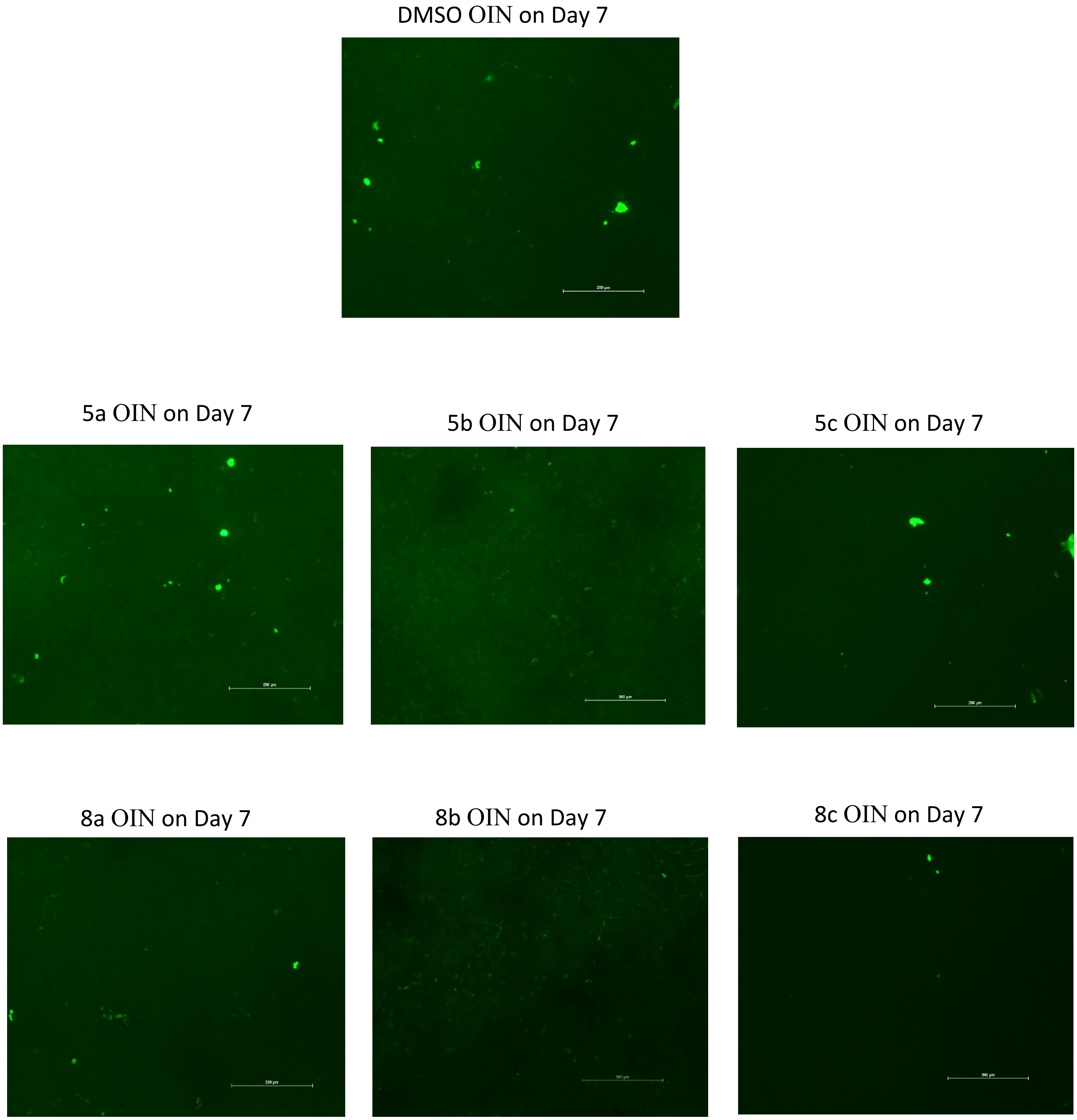

Supplement: RA-008-C8RA00646F-s001 [file RA-008-C8RA00646F-s001.pdf]
